# Roles, relationships, and motor aggressions: Keys to unveiling the emotions of a traditional sporting game

**DOI:** 10.3389/fpsyg.2023.1127602

**Published:** 2023-01-30

**Authors:** Pere Lavega-Burgués, Verónica Alcaraz-Muñoz, Carlos Mallén-Lacambra, Miguel Pic

**Affiliations:** ^1^Group of Research in Motor Action (GIAM) National Institute of Physical Education of Catalonia (INEFC), INDEST, University of Lleida, Lleida, Spain; ^2^Group of Research in Motor Action (GIAM), Facultad de Educación, Universidad Católica de Murcia (UCAM), Murcia, Spain; ^3^Group of Research in Motor Action (GIAM), Institute of Sport, Tourism, and Service, South Ural State University (SUSU), Chelyabinsk, Russia

**Keywords:** coexistence, motor praxeology, quality education, sustainability development, affectivity, traditional game, physical education

## Abstract

International organizations such as the UN and UNESCO set priority goals for education in the 21st century. This article shows the educational contribution of the Traditional Sporting Game (TSG) of Bear Guardian and Hunters that involves the three-chained roles. The three roles test players who share a unique social interaction ritual. This study was part of a training experience for university students in physical activity and sports sciences in the theory and practice of motor games subject at INEFC, University of Lleida (Spain). This research investigated the emotional intensity in these three roles, the emotional meaning units, and their correspondence with the emotional triad. This study is a mixed-methods research. After playing the game involved, 131 university students (46 women and 85 men) aged 18–35 years (*M* = 20.19, *SD* = 2.42) answered the validated GES-II scale indicating the intensity and causes of five basic emotions. The data were analyzed using different strategies (qualitative data: content analysis; quantitative data: descriptive statistical analysis, inferential and association rules). The methodology employed has revealed part of this game’s secret (intimate and subjective) code: the affectivity invisible to external observation. Among the findings, we highlight: (a) each role originates different intensities and units of emotional meaning; (b) the three roles feedback, need and complement each other in this socio-affective network of interdependent relationships; (c) the Bear is the central role of the game. The emotional meanings concerning the motor aggression of the Bear operate as a magnet that attracts four itineraries of association rules of meanings and emotional triads. In the hands of intelligent, prepared and sustainable teachers, this game can help students learn to live together and educate them to control and respectfully channel motor aggression. In this way, students will be active actors in the process of civilization in favor of sustainable development.

## Introduction

### Social interaction as ritual

Since human beings are born, we learn to live with other people in different interactive contexts. Living in society implies daily participation in rituals of interaction that take place on a small scale, here and now, face to face, where the relationship builds people as social subjects (Goffman, 1983; Collins, 2009).

According to Goffman (1983), social relations establish a ritual order. Thus, in any social interaction, actors perform some Role. This performance or enacted Role is addressed to the other interaction participants and potential observers. Social interactions represent the roles that each actor has internalized in such a way that they form part of their own identity.

Roles play a fundamental role in the interaction between people, indicating the type of conduct their actors are expected to carry out in a given situation.

When interacting with others, the person shows a specific type of information about him/herself, depending on the situation and the intention, which will provoke different responses depending on how others interpret him/her. As in a theater, there are pre-established behavioral limits in every interaction, a script to be interpreted in front of others.

This ritual order makes sense in the social and cultural context in which it takes place. Thus, in the rituals of interaction, people intervene following the social norms of their community or society. Furthermore, in any social interaction, a ritual is created that organizes and orders the relationships between people and how they express and manage their emotions. The flow of emotional energy that actors share (when emotions enter into reciprocal consonance) is a central ingredient and outcome of the interaction ritual (Collins, 1984, 2009).

From this shared emotional energy emerge symbols of social relatedness that evoke a sense of belonging to a group. People interact in an affective encounter with themselves, others, and their environment in everyday life. Thus, emotional literacy takes place, which favors the civilizing process of self-control of emotions in a modern and complex society such as ours (Elias, 1987).

In this context of emotional literacy and self-control, interpersonal relationships are governed by two interaction rituals: power and status, which have a specific emotional energy. According to Kemper (1981), in power interactions, there is an expectation to dominate others, whereas, in status relationships, one seeks an exchange of relationships to satisfy his or her own needs, as well as other participants’ needs and wishes.

Some power interactions can give rise to interpersonal conflicts and even to aggression or physical violence involving the use of force (Elias and Dunning, 1994).

Interaction rituals facilitate their actors’ social and emotional literacy, channeling violent and aggressive behaviors into cordial and respectful relationships. According to Goffman (2004), the order of social interaction is sustained by a moral order that is constituted around care, protection and respect for the members who participate and are recognized in this social system. Behavior must be honorable, dignified and respectful in a social encounter. Failure to meet these expectations implies a deviation from this moral order (Goffman, 2004).

### Challenges in the 21st century to educate sustainable rituals of interaction

A straightforward way to identify the priorities for education in the 21st century is to look at the guidelines set by such representative international organizations as the UN and UNESCO. Irina Bokova, Director-General of UNESCO, indicated a few years ago that “now, more than ever, education has a responsibility to be attuned to the challenges and aspirations of the 21st century and to foster the right kinds of values and skills that will lead to sustainable and inclusive growth and peaceful coexistence” (UNESCO, 2016, p. 6).

Thus, the challenges of all modern education tend to align with the Sustainable Development Goals (SDGs) adopted by United Nations (UN) (2015), an ambitious and universal agenda to transform the world. Physical education should also orient its priorities toward Education for Sustainable Development (ESD), seen as a critical instrument for its achievement:

- *SDG 3: Good Health and Well-being* related to ensuring healthy lives and promoting well-being for all ages. In this goal, the learner can encourage others to decide and act to promote health and well-being for all.
- *SDG 4. Quality Education to ensure inclusive and equitable quality education and promote lifelong learning opportunities for all*. The learner understands that education can help create a more sustainable, equitable and peaceful world.

Through these two objectives, students should learn to participate in rituals of interaction that trigger socio-emotional experiences of health, well-being, sustainability, equity, and peace.

Thus, sustainable development education has to develop competencies that empower students to reflect on their actions, considering their current and future social and cultural impacts from a local and global perspective. Sustainability students should engage constructively and responsibly with today’s world. According to UNESCO (2016), the learning of SDGs includes cognitive, socioemotional and behavioral elements. Hence, they are an interplay of knowledge, capacities and skills, motives, and affective dispositions.

### Physical education in the 21st century, learning to live with new rituals: Sustainable motor interactions

A careful reading of the foundations established by the Education for Sustainable Development Goals (United Nations (UN), 2015) has made it possible to identify learning objectives, competencies, procedures and other priority guidelines to which modern physical education should respond (Niubò-Solé et al., 2022).

The architecture of this physical education for sustainable development should be built on three pillars:

- Quality education. PE has to be understood as an integral part of quality education.
- Educate sustainable social relationships: health and socio-emotional well-being oriented toward an equitable and peaceful world.
- Action and reflection in action. Competencies are acquired during action based on experience and reflection. They activate cognitive, socio-emotional, and behavioral domains.

To respond to these challenges with solvency, a scientific discipline is needed to generate evidence and apply scientific knowledge. It is necessary to promote physical education based on scientific evidence. The theory of motor action or motor praxeology offers a new vision, a paradigm shift, by conceiving physical education as a pedagogy of motor behavior (Parlebas, 2001). From this perspective, the student is the center of attention who, by participating in a game, sport or physical exercise, activates his or her whole personality in a unitary and systemic way.

To educate motor conduct, the trainer has a wide range of educational resources (games, sports, exercise) that will test the students in different motor interaction rituals.

According to motor praxeology, each motor practice has a singular internal logic which requires the student to have a specific way of relating to the other participants, to space, to objects and to time (Parlebas, 2001).

Each game triggers a frame of meaning (Goffman, 2007) in which players engage in contextualized motor conduct (Parlebas, 2001). This context of meaning corresponds to the implementation of rules understood as a set of rights and prohibitions that players have decided to accept. This is how each game or sport originates a singular motor interaction ritual that will elicit motor conduct of a different nature.

The concept of motor conduct refers to “the meaningful organization of the actions and reactions of a person who acts, the relevance of whose expression is motor in nature” (Parlebas, 2001, p. 85). Motor conduct goes beyond the mechanistic and decontextualized view of the movement to refer to the unitary intervention of the person. This concept is particularly noteworthy; it relates to the person as a unique, singular being which expresses all its dimensions when engaged in organic, cognitive, emotional and relational levels (Parlebas, 2001).

The traditional sporting game (TSG) deserves special attention among the possible pedagogical resources. The rules are linked to the local culture, and, unlike sports, they are not governed by an institution or federation (they are not institutionalized).

The social and interactive nature of the TSG rules activates a unique web of symbolic relations between the players (Geertz, 2003). The originality of this symbolic ritual is that it corresponds to lived culture, played and expressed through motor actions (Parlebas, 2001). We are dealing with a lived intangible cultural heritage, that is to say, in the case of the traditional game, with a played intangible cultural leisure heritage.

The rules of the TSG are authentic showcases, repositories of the values and the type of motor interaction representative of the community that hosts them. They are a strong vector of socialization, behaving like miniature societies (Parlebas, 2000) that give life to a wide variety of rituals of motor interaction.

The TSG are frames of the meaning of immediate physical presence, where people express a vision of the situation, of others and themselves. “The positive social value a person effectively claims for himself by the line others assume he has taken during a particular contact” (Goffman, 1970, p. 5).

The originality of the TSG lies precisely in the diversity of their motor interactions. Sometimes a player plays alone (skittles), while in other games, he or she cooperates with more than 500 people (human towers). Some games that pit two people against each other (wrestling games) or two teams (such as Dodgeball), just like Olympic sports.

The TSGs maintain original rituals of motor interaction that are not present in the interactive structure of sports. There are games in which people can change teams during the game (e.g., a chain game in which, in the beginning, two people chase each other, and at the end of the game, they all go together against the last player). In some paradoxical games, players are potential partners and opponents of each other (e.g., the four corners in which corner players can ally or betray each other as they wish; Lavega-Burgués et al., 2022).

Learning to live together with others involves sharing relationships and emotions in a wide variety of motor interaction rituals, which the TSG also offers. In this ludomotor plot, the biological nature of emotions is harmonized with the social nature of play (Kemper, 1981). The affective subjectivity of each actor (motor conduct) is intertwined with the objective social situation offered by the rules of any game (internal logic). Moreover, in the case of the TSGs, as they are rules of local tradition, the emotions carry the meaning of the social norms of their community. They are “feeling rules” (Hochschild, 1979) that define what it is appropriate to feel in each of these rituals of social relations (for example, to be happy when our team wins, even though we have not played well).

In this ritual of motor interactions, emotional responses depend on each person’s interpretation of the motor situation in which he or she is involved (internal logic). Emotions also respond to the meaning that originates from the exchange of interpersonal signs (motor behaviors) with the other participants (Lavega et al., 2014).

“A group microculture emerges in this interpersonal exchange of signs, which generates and transmits a system of norms, values and common ways of doing things to establish a cognitive, relational and affective network of shared socio-motor meanings” (Parlebas, 2016, p. 178).

TSGs correspond to frames of meaning (Goffman, 2007), i.e., contexts loaded with social meanings that give sense to the actions and symbols transmitted by their actors. According to the culture, there are different ways of interpreting the social world, the symbols and the rituals that each actor must follow in this social interaction. In this way, each TSG originates a web of ritualized socio-affective motor interactions.

The TSG players participate in living and real procedural learning in this ritualized weft. Each person is free to orient their motor interactions toward a relationship or ritual of power or status interaction. Moreover, in some cases, the rules authorize the exchange of intense motor interactions, as in the traditional game of Bear-Guardian and Hunters.

These games become potent educational resources whose potential can educate interpersonal relationships in a playful context. They can regulate physical violence by transforming it into a respectful and peaceful socio-emotional encounter with all participants.

### The traditional sporting game of Bear, Guardian, and Hunters. A ritualized socio-affective interaction

The Bear and the Guardian is a traditional game played in different times and societies and is known by various names. Parlebas (2000, p. 8), when studying this game, noted the following denominations: Game of the pivot (paintings of Herculaneum first century after J.C.); game of the nail (Rome, III century after J.C.); the devil in chains in the painting of Brueghel (Antwerp, 1,560); The Poira of Jacques Stella (Paris, 1,657).

The Bear and the Guardian is a game with apparently simple rules that, in reality, gives rise to relatively complex social relations.

### The internal logic of the Guardian, Bear and Hunters. The chained role reversal

Analysis of the internal logic of this game identifies three roles associated with different rights and prohibitions that players must obey.

#### The Bear

A player sits on the ground and is attached to the Guardian by a rope. It is a passive role, as he cannot intervene to avoid Hunter’s blows or help the Guardian.

#### The Guardian

This player takes on this role and connects with the Bear by the end of a rope that he/she cannot release. He/she engages in negative motor interactions (opposition) toward the hunters, whom he/she tries to hit with a handkerchief in one of his/her hands. The Guardian directly opposes a hunter who threatens the Bear and protects the person in the role of Bear through indirect cooperation or positive motor interaction. This interaction is a ternary relationship in which the cooperation of the Guardian with the Bear takes place through the adversary (hunters).

#### The Hunters

The rest of the players take the role of the Hunter and carry a handkerchief in their hand to hit the Bear’s body without the Guardian touching them. Tactical alliances and complicities may arise between the Hunters, but the rules do not establish a formal relationship of solidarity between these players (Parlebas, 2000).

**Figure 1:**
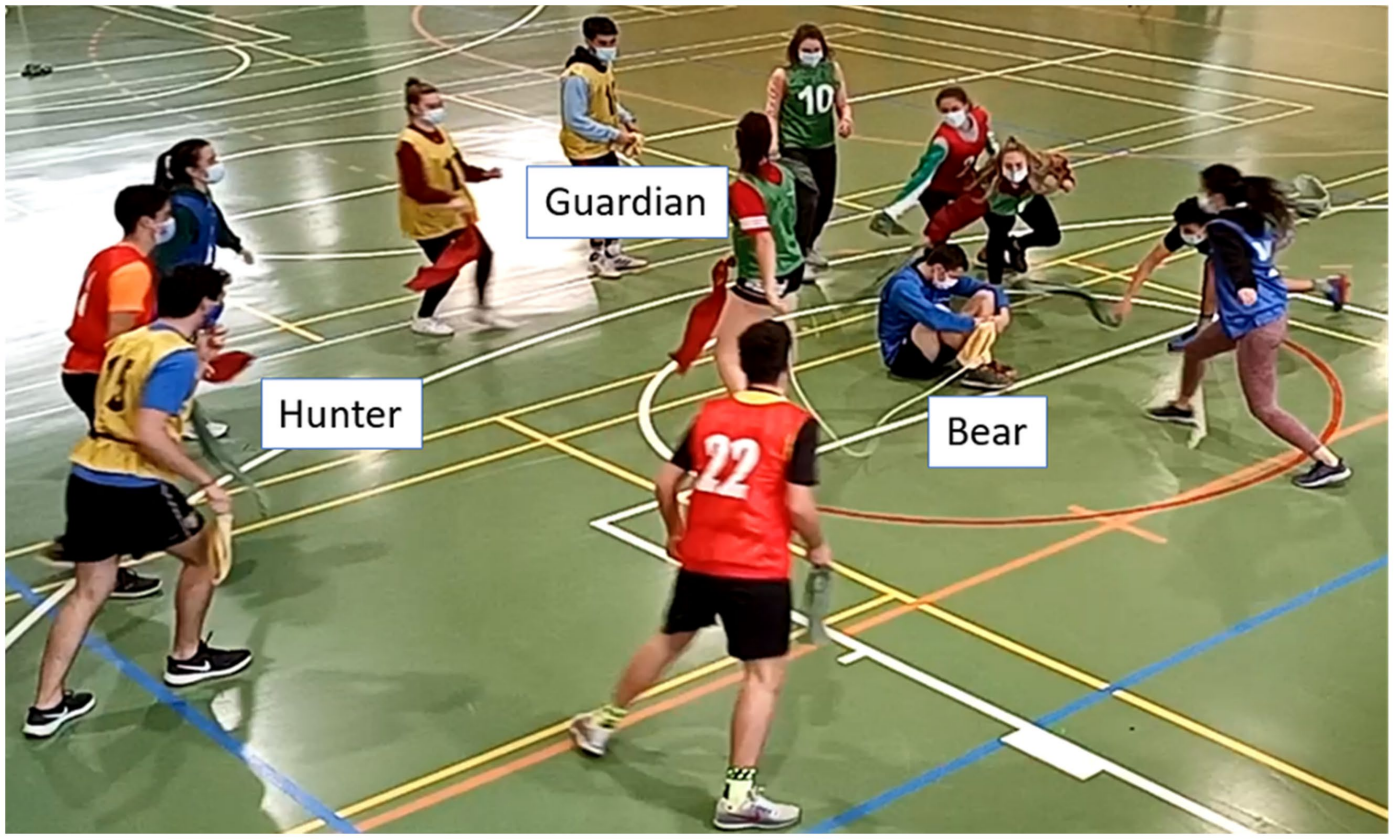
The roles in the Bear-Guardian and Hunter Game.

When a Hunter is hit with a handkerchief by the Guardian, that player switches to the Bear role, the Bear moves to the Guardian role, and the Guardian changes to the Hunter role (see Figure 1).

Each role change establishes the end of a sequence of play. The game is a succession of role reversals simultaneously, as this triad is affected by a chained permutation between the Hunter, the Bear and the Guardian. The internal logic favors that the participants can go through all the roles of the game, giving rise to a web of complex social interactions.

Unlike Olympic sports, the rules of the game do not establish a way to end the game. Tiredness, the start of another activity or any other external criteria can be the reason for ending the game. Moreover, this criterion may vary depending on who the players are or the game’s conditions.

We are dealing with a small social universe, which gives rise to a singular motor interaction ritual based on an original system of interpersonal motor relations.

### The motor conducts of the Bear, Guardian, and Hunters in a chained plot of relationships and emotions

This game activates the dynamism of motor conduct with a strong emotional charge. At the same time, it triggers solidarity and physical aggressiveness, which makes possible the constant adaptation of the players according to the chained change of the three roles (Parlebas, 2000, p. 8).

The rules of this game authorize Hunters’ motor aggression on the Bear, as they can hit its back with a handkerchief. We can refer to a lawful motor aggressiveness that corresponds to those motor interactions between opponents whose motor conduct is authorized by the rules (Collard, 2004; Collard and Oboeuf, 2007; Dugas, 2008). On the other hand, players may engage in other motor conducts associated with illicit motor aggressiveness or physical violence when their intervention is harmful and affects the physical integrity of other people (e.g., punching an opponent). Such violence is illegal and, therefore, punishable.

In the past, traditional games incorporated a high degree of motor aggression in the participants’ interactions (e.g., soule as a predecessor of today’s rugby), who risked getting hurt in the matches. Over time, society has civilized the intensity of interpersonal relations. So now, the conflict between two sides is ritualized in a web of regulated motor interactions, as a sport so well represents. It is the process of civilization of society and of traditional sporting games (Elias, 1987).

The practice of this game does not eliminate aggressive behavior but allows the emergence of aggressive behavior and enables each student to learn to channel such conduct toward a noble, respectful and peaceful coexistence objective (Loyer et al., 2015). The player can assess the motor aggressiveness of his or her strokes (moderate or intense motor aggressiveness, Collard, 2004) and become aware of the emotional consequences (e.g., fear, sadness, anger, and rejection).

In the set of motor interactions with different levels of motor aggressiveness, collective emotions play a pivotal role in shaping players’ responses to conflicting events and in contributing to the evolvement of this social interaction that maintains the emotional climate and collective emotional orientation that they have developed (Bar-Tal et al., 2007).

The Bear, Guardian and Hunters game originates a network of motor interactions ordered under a cycle of ternary permutation between the three roles. In each Role, people can make different decisions and test their socio-motor empathy and the management of their emotions for each Role. Each of the three roles carries different ways of relating and experiencing emotions.

Based on the theoretical framework and the explanations of the contribution of traditional games in general and the Bear-Guardian-Hunter game in particular, this research aimed at the following objectives.

1. To identify whether there are significant differences in the emotional intensity experienced by players in the roles of Bear, Guardian, and Hunter.
2. To recognize the units of emotional meaning arising from participation in Bear, Guardian and Hunter roles.
3. To reveal the correspondence between the positive or negative emotion experienced in the three roles (emotional triad) and the emotional meaning triggered by the Bear, Guardian and Hunter motor interactions.

## Method

### Design

This research corresponds to a mixed-method study. It contains qualitative and quantitative results that have been integrated to ensure the information mixing (Teddie and Tashakkori, 2010; Anguera et al., 2018).

Initially, the research corresponds to a qualitative, descriptive and interpretative study carried out in natural conditions (Miles and Huberman, 1994; Taylor and Bogdan, 2000; Krippendorff, 2002). In the absence of previous studies on the emotional meaning of motor interactions in this game, it has been necessary to describe the findings (units of emotional meaning in the three roles according to different phases of content analysis of reduction, separation and grouping). It is naturalistic since the experience was conducted under normal conditions, where university students usually participate in practical sessions.

In parallel, the hermeneutic units were arranged in a database to be statistically analyzed (quantizing qualitative data) according to an associative strategy (exploring the functional relationship between variables; Ato et al., 2013). In this case, we explored the statistical relationship between the different hermeneutic units (role, motor interaction and motor aggression) and the emotions experienced in the three roles (emotional triad).

Finally, the study is interpretative in that the results obtained in the content analysis and the quantitative analysis have been interpreted in a mixed methods manner by the theoretical framework of reference (fundamentals of motor praxeology linked to the interaction approach as ritual).

### Participants

A total of 131 university students (46 women and 85 men) aged 18–35 years (*M* = 20.19, *SD* = 2.42) on the undergraduate degree in Physical Activity and Sport Science offered by the National Institute for Physical Education of Catalonia (INEFC) at the University of Lleida, Spain, took part in the study. The IRB/Ethics Committee approved the study and Clinical Research (CEIC) of the University of Lleida, and all participants gave their consent to participate.

### Instrument and procedure

The 131 participants in this study carried out the intervention in five groups of 25 to 35 people, according to the usual organization of the practical sessions of the subject. We followed the indications described by the CEMEA group in France (Parlebas, 2001) in describing how to carry out this game. According to the rules, at least eight players and a maximum of 13 people can play. In each group, the participants were distributed in two or three zones (groups of up to 26 students in 2 zones; groups of more than 26 people in three zones).

The players participated in this game for 8 min since this is the duration used by the motor action research group (GIAM) in previous studies on other traditional sports games.

After finishing the game, each participant individually answered the validated questionnaire on sports games and emotions GES-II (Lavega-Burgués et al., 2018). This questionnaire expressed the intensity (Likert scale from 1 to 7) experienced in five basic emotions (joy, anger, fear, sadness, rejection). For the emotion registered with the greatest intensity, the reason for that intense value was requested to be described.

### Statistical analysis of quantitative data

Descriptive statistics, mean and standard deviation, were calculated for positive and negative emotions by roles. The effect sizes (E.S.) interval of >0.2 small, >0.5 moderate and > 0.8 large were used to interpret the differences calculated according to recommendations (Cohen, 1988).

### Content analysis of qualitative data

The qualitative data collected with the GES-II questionnaire were analyzed using the “content analysis” technique to formulate valid, applicable inferences according to the context of the study (Krippendorff, 2002). The analysis enabled categories to be formed using the method developed by Miles and Huberman (1994), which consists of three phases: (1) to reduce, (2) to separate, and (3) to synthesize and group units of socioemotional meaning.

#### Reduction

Once the comments were transcribed verbatim, the reduction of data into units of meaning was first made deductively, following the theoretical approach of reference. Three significant units were observed for each Role of Bear, Guardian, and Hunter:

- Role. This unit corresponded to comments referring to the role in general, to the strategies used, without making any explicit comment on the motor interaction (e.g., I felt joy because the role of Hunter was very dynamic; I felt fear because I did not think that I would not change roles; I felt joy because thanks to my strategy I was able to change from the role of Guardian to Hunter).
- Motor interaction. Comments from this unit referred to cooperative or opposed interactions between the roles (e.g., I was happy that I quickly captured a hunter; I felt sad that the Guardian did not protect me; I felt terrible being captured by the Guardian).
- Motor aggression. In this unit, the comments described an intense motor interaction between the players associated with motor aggressiveness (e.g., Playing the Role of Bear was not a problem for me, despite receiving some strong blows from the Hunters; I was amused to see the Bear’s reaction when he screamed and also laughed when receiving a strong blow to his back).

#### Separation

Suddenly, through an inductive procedure, the units of meaning were separated so that they could be synthesized for each Role. The units referring to Role, Motor Interaction and Motor Aggression were separated according to two criteria. The first criterion of type of emotion originated two large groups of testimonials referring to emotional well-being (positive emotion of joy) and emotional discomfort (negative emotions, fear, sadness, rejection, anger). The second criterion corresponded to the direct or indirect relationship with the registered unit. They separated into a first group that showed a direct relationship with the observed unit of meaning (e.g., I had a good time spending a short time in the Role of Bear). In the concept map, we identified them with the proposition FOR. The second group corresponded to explanations indicating feeling an emotion despite being in an undesirable situation (e.g., I was fine in the role of the Bear despite having been beaten by the Hunters; I liked the role of the Guardian very much, even though I could not prevent the Hunters from attacking the Bear). In these cases, we identified the comments in the concept map with the expression DESPITE.

#### Synthesizing and grouping

**Figure 2:**
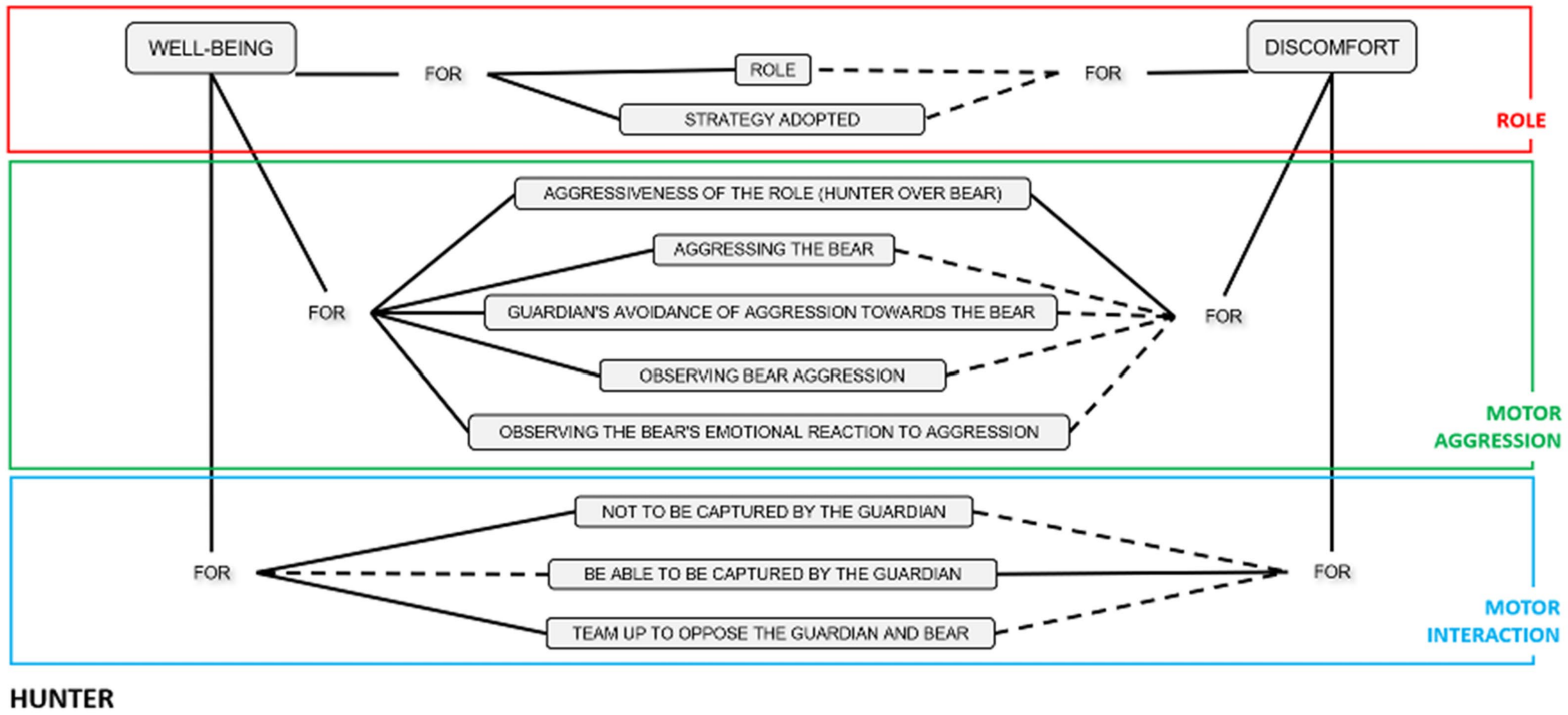
Units of emotional meaning in the Bear role.

**Figure 3:**
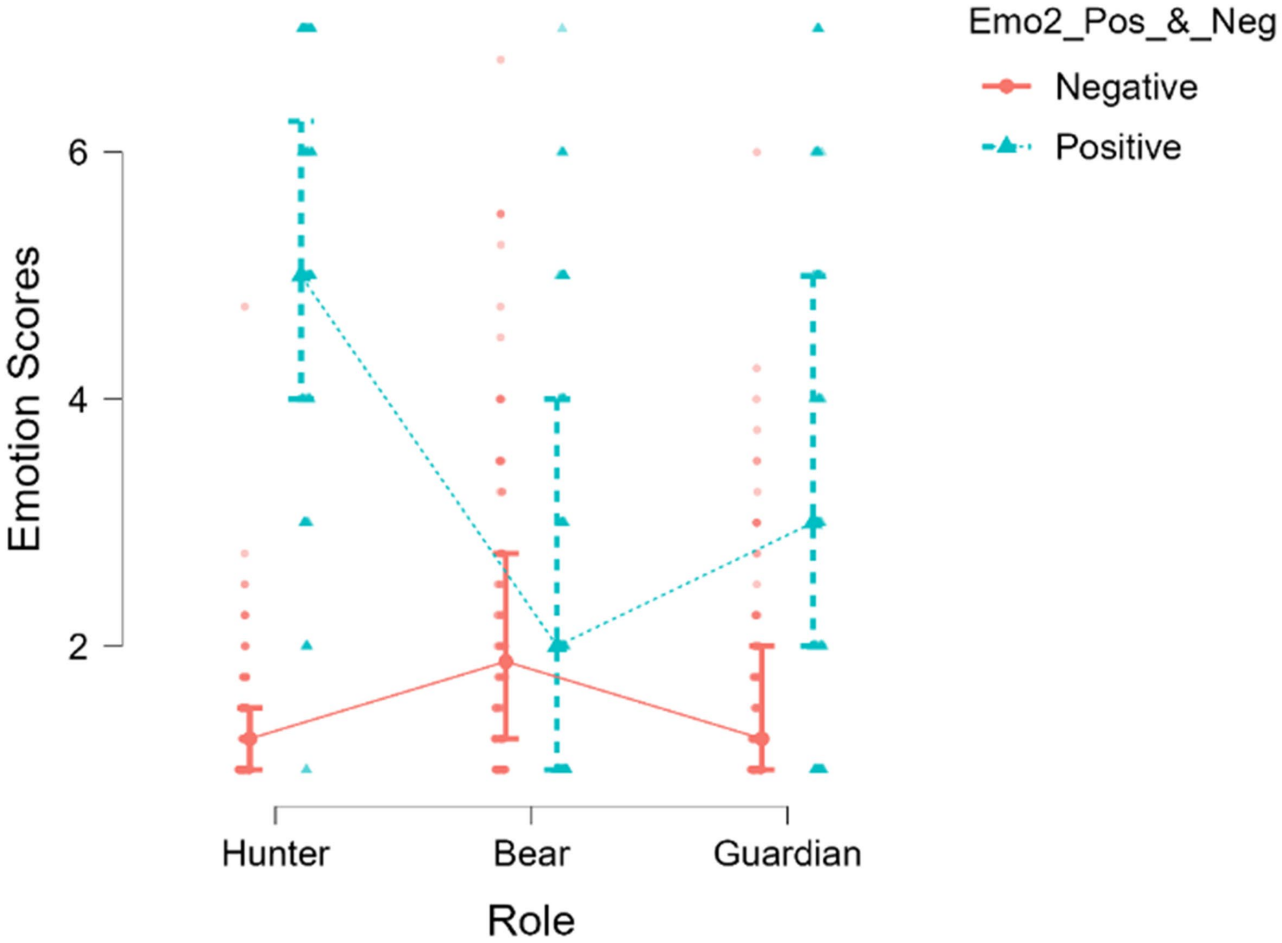
Units of emotional meaning in the Guardian role.

**Figure 4:**
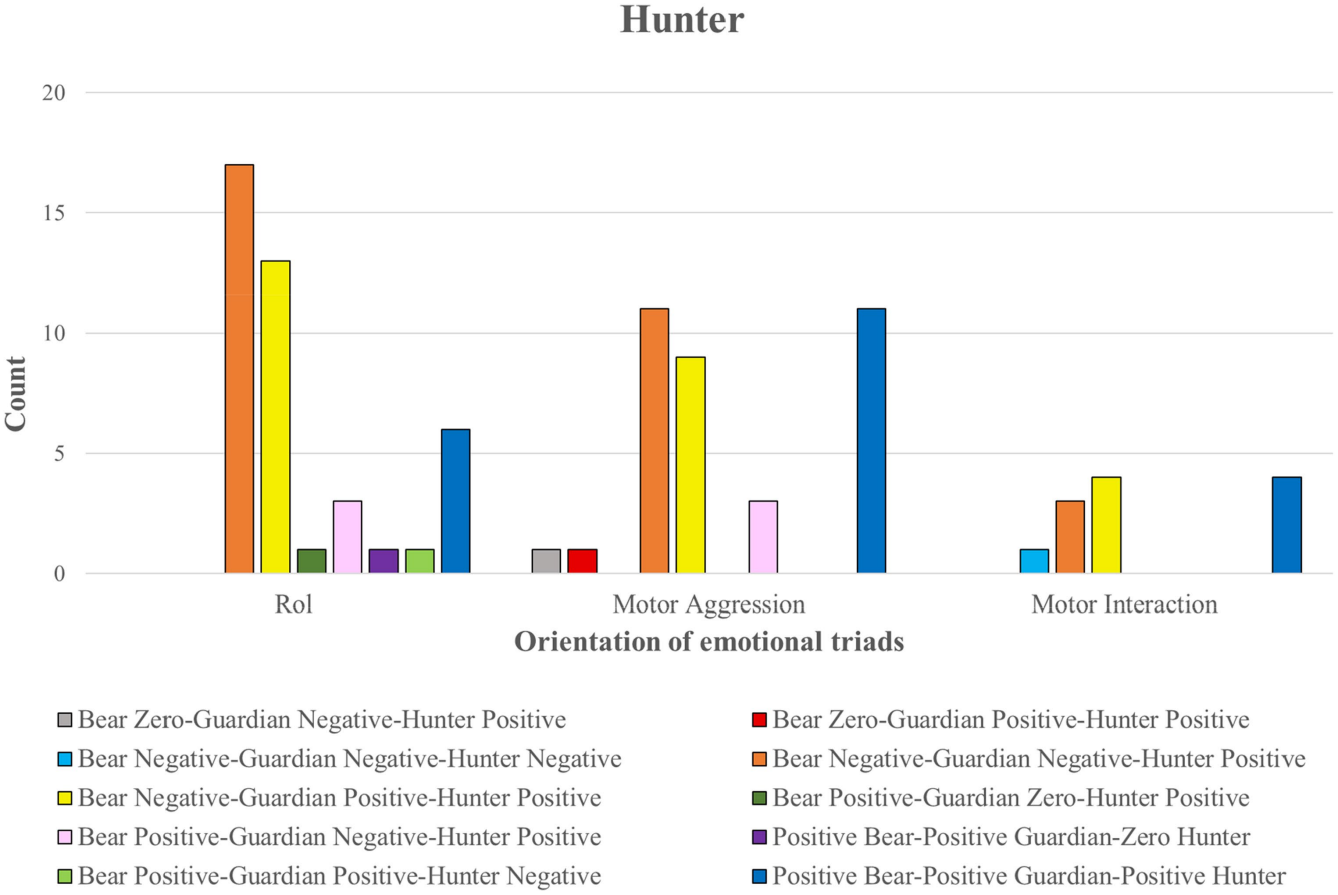
Units of emotional meaning in the Hunter role.

Finally, we group each Role’s meaning units in a concept map. We drew a broken line to express that although this option was not observed, these emotional meanings could be given to other groups of people. In the other cases, the continuous lines showed that this unit had been registered with the participants of this study (see Figures 2–4).

### Statistical analysis of qualitative data

For the present study and the purpose of analysis, descriptive and inferential data analyses through crosstabs (Luchoro-Parrilla et al., 2021; Ormo-Ribes et al., 2021) were computed. The Pearson’s Chi-square test and Cramer’s V (effect size) (Field, 2013) were performed to compare the obtained frequencies according to (Oso_orientacion_emociones/Guardian_orientacion_emociones/Oso_orientacion_emociones/Triada Emo2) with special attention to adjusted residuals (ARs) > 1.96 or < −1.96. The significance level was set at *p* ≤ 0.05.

The statistical package SPSS version 25.0 (IBM Corp., Armonk, NY, United States) was used for the analyses. On the other hand, RapidMinner 9.10 was used to uncover association rules between the study variables. The measures observed in the results were support, confidence and lift (Park et al., 2014). Support refers to how often a given rule appears in the database (Support = Freq (*X*, *Y*)/*N*). Confidence measures how often each item in Y appears in an interaction that contains items in *X* also (Confidence = Freq (*X*, *Y*)/Freq(*X*)). Finally, the lift value (between 0 and infinity) is a measure of the importance of a rule (Support/Supp(*X*) * Supp(*Y*)). A lift value greater than 1 indicates that the rule appears more often together than expected. A minimum confidence value was set at (<0.4) to select the association rules.

**Figure 5:**
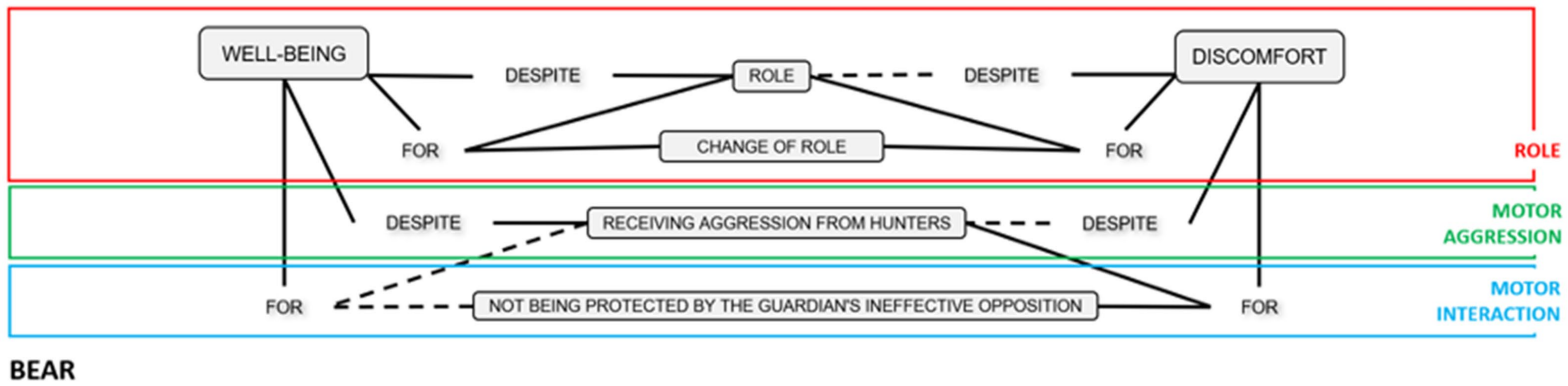
Itinerary of the emotional meanings in the three roles. The thickness of the lines corresponds to the frequency of comments. The thicker the lines, the more comments there are.

**Figure 6:**
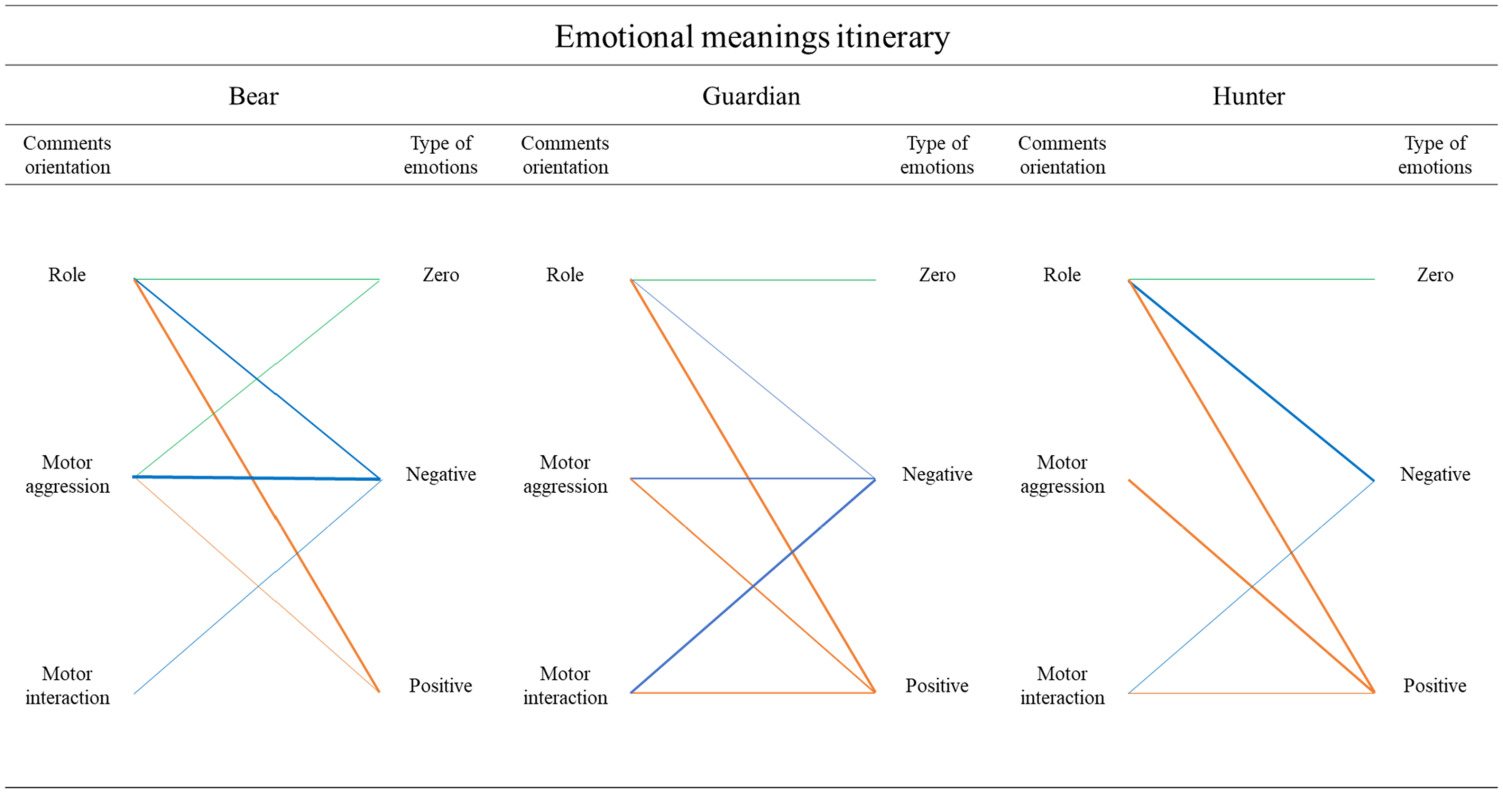
Emotional itinerary in the chained roles. The thickness of the lines corresponds to the frequency of comments. The thicker the lines, the more comments there are.

Figures 5, 6 reflect the intensity of the relationships between variables. While thin or missing lines mean a weak relationship, thicker lines reveal a stronger relationship.

### Methodological integrity

The methodology followed has considered the guidelines of Levitt et al. (2018) referred to the Journal Article Reporting Standards for Qualitative Primary, Qualitative Meta-Analytic, and Mixed Methods Research in Psychology. The claims made from the analysis are warranted and have produced findings with methodological integrity. The study followed procedures that supported methodological integrity.

#### Adequacy of the data

The data was obtained from the comments described by the participants seconds after finishing the game. Each student answered separately to avoid interference. It was made clear that there were no right or wrong answers but that they were different and corresponded to the emotional meaning each person had given to their motor conduct.

#### Grounded findings in the evidence

The findings correspond to the analysis of the students’ texts in their literal versions. The procedure followed is based on evidence obtained in previous studies, some of which were doctoral theses published in various scientific articles of impact. All this has allowed this study to move forward to reveal the phenomenon of the emotional meaning of the game, which is invisible to any observer.

#### Data quality control

The content analysis followed a rigorous procedure among three researchers that lasted 6 months. Three university students in physical activity and sports sciences with a master’s degree in physical education, experts in the discipline of motor praxeology and previous studies on content analysis of emotional states in other games participated. The 90 comments were arranged in a database in excel format. Subsequently, each text was associated with one of the five emotions. Those researchers took part in 40 h of training on content analysis following the guidelines of Anguera and Blanco (2003). This training made it possible to prepare a reference manual in which the criteria and components that each unit of emotional meaning could include were described.

The manual was the result of (1) reducing, (2) separating, and (3) synthesizing and grouping units of emotional meaning. The units of meaning created in the manual were reviewed several times in a deductive and inductive way by the three researchers until a final version was obtained. Subsequently, the three researchers performed a pooled analysis of the first 50 comments. Then, each researcher separately analyzed the following 50 comments. Then, possible disagreements were met and discussed until the result of this analysis was unified, guaranteeing complete inter-rater agreement. This procedure was repeated until the analysis of all comments was completed. The units of meaning created in the manual were reviewed several times in a deductive and inductive way by the three researchers until a final version was obtained. Subsequently, the three researchers performed a pooled analysis of the first 50 comments. Then, each researcher separately analyzed the following 50 comments. Then, possible disagreements were met and discussed until the result of this analysis was unified, guaranteeing complete inter-rater agreement. This procedure was repeated until the analysis of all comments was completed.

Cohen’s kappa coefficient was applied to measure the level of inter-observer agreement (stability and objectivity). The values ranged from 0.86 to 0.91 (in the first 50 content analysis) and 0.90 to 0.96 (in the other content analysis).

## Results

### Emotional intensity in the Bear, Guardian, and Hunter roles

The statistical analysis showed that in the three roles, the positive emotions were more intense than the negative emotions (see Table 1). The size of Cohen’s effect *d* (ES = Effect Size) of the positive emotions concerning the negative emotions reached high values in the Hunter (ES = 3.09) and Guardian (ES = 1.18) roles. This size was significantly smaller in the Bear role (ES = 0.39).

**Table 1:** Positive and negative emotions (mean, standard deviation, and effect sizes) by roles.

Intra-role	Positive emotions	Negative emotions	Effect size (Cohen’s *d*)
Bear	2.77 ± 1.72	2.17 ± 1.25	Positive > Negative = 0.39
Guardian	3.41 ± 1.89	1.66 ± 0.88	Positive > Negative = 1.18
Hunter	5.09 ± 1.61	1.38 ± 0.53	Positive > Negative = 3.09

Positive emotion intensities between roles were also compared. The Hunter role elicited higher values of joy (*M* = 5.09; SD = 1.61) than the Bear role (*M* = 2.77; SD = 1.72) (ES = 1.39 Hunter > Bear). The Hunter role also activated stronger values of Joy than the Guardian role (*M* = 3.41; SD = 1.72) (ES = 0.95 Hunter > Guardian). The values of joy were also more intense when comparing the Guardian role with the Bear role (ES = 0.35 Guardian > Bear).

Comparing the intensity of negative emotions also found significant differences between the three roles. The Bear role produced more intense negative emotions (*M* = 2.17; SD = 1.25) than the Hunter role (*M* = 1.38; SD = 0.53) (ES = 0.82 Bear > Hunter). The role of Bear also raised either higher negative emotion values than the Guardian role (*M* = 1.66; SD = 0.88) (ES = 0.47 Bear > Guardian). Finally, indicate that negative emotions were more intense in the Bear and the Guardian roles for those who originated in the Hunter role (ES = 0.38 Guardian > Hunter).

**Figure 7:**
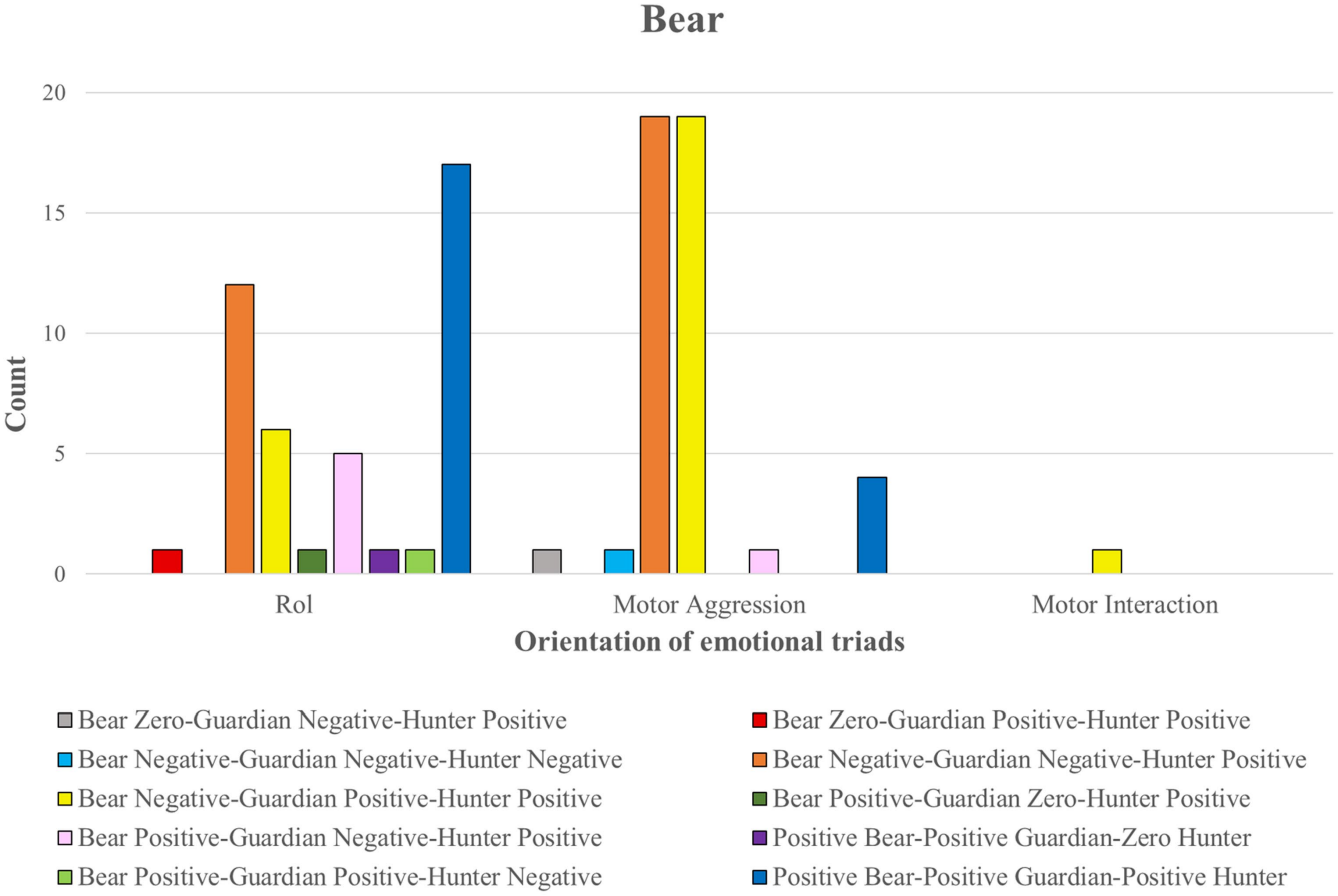
The intensity of positive and negative emotions in the Bear, Guardian, and Hunter roles.

Figure 7 shows the behaviors of the three roles in positive and negative emotional states.

This study explored the emotional meaning attributed to the three roles to provide quality in the findings of the statistical treatment. The content analysis of the students’ comments identified three main dimensions of emotional meanings in each of the roles, Bear, Guardian and Hunter, referring to (a) the role, (b) motor interaction, and (c) motor aggressiveness. The following sections use concept maps to describe the units of emotional meaning (of well-being or discomfort) for each of these three main dimensions. In the conceptual maps, the continuous lines correspond to the units observed with the participants studied. In contrast while the dashed lines refer to categories that might appear if this study were carried out with other participants.

In a second section, for each role, we show the relationship between the three significant dimensions of emotional meaning (role, motor interaction and motor aggressiveness) and the emotional triads that arise from participation in the three chained roles of this game (Bear: Zero ZB = without an answer; PB = Positive Emotion; NB = Negative Emotion; Guardian: Zero ZG = without an answer; PG = Positive Emotion; NG = Negative Emotion; Hunter: Zero ZH = without an answer; PH = Positive Emotion; NH = Negative Emotion).

### Units of emotional meaning in the Bear role

The role of the Bear mostly gave rise to negative emotions, although some people also highlighted the emotion of joy.

Players in the role of Bear expressed discomfort at not being able to change roles. On other occasions, emotional well-being was felt when the role change occurred quickly. The discomfort could also be due to not being protected by the ineffective strategy of the Guardian. Likewise, players in the Bear role expressed discomfort when receiving motor aggression from the Hunters. Surprisingly, some people expressed joy in the game, despite receiving aggression with the hunters’ handkerchief.

### Relationship of emotional meaning with the emotional triad in the Bear role

The 90 comments on the role of Bear included testimonies related to the emotional meaning of the role (*n* = 45) and motor aggression (*n* = 44). In contrast, only one comment was associated with the emotional meaning of motor interaction.

**Figure 8:**
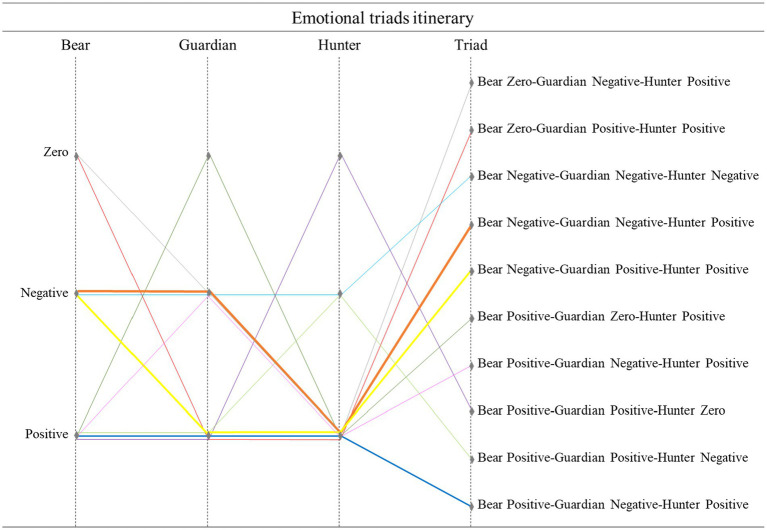
Orientation of emotional triads in the Bear role.

Three triads stand out from the 10 possible emotional triads due to the positive or negative emotion in each of the three roles (see Figure 8). Two triads with negative emotions for the bear ON_GN_CP (orange color) (*n* = 31 comments), ON_GP_CP (yellow color) (*n* = 26 comments); and one triad with positive emotions for the three roles OP_GP_CP (dark blue color) (*n* = 21 comments).

The two bear well-being triads corresponded to comments associated with the emotional meaning referred to the role (testimonies expressing joy at being able to change from the role of Bear to another more favorable role). Emotional triads linked to a negative experience of the Bear were frequent in comments oriented to the emotional experience of motor aggression.

The cross-table statistical test relating the three types of comments (role, motor interaction and motor aggression) to the emotional triads found no significant differences (*p* = 0.069; *Cramer’s V* = 0.39). However, some trends of interest were observed in three emotional triads.

The triad OP_GP_CP (dark blue color) was represented by 21 comments referring to the emotion of joy in the role of Bear. Most of these comments (*n* = 17; *residual fit* = 3.4) referred to the role. Relating these results to the emotional map shown above (see Figure 8), it can be deduced that these are emotional states of well-being elicited by being able to change roles. On other occasions, despite being in this role, players report feeling joy.

*“It was a fun role; even though we got beaten up, we were laughing, and so I had a good time”.C1*

We identified four comments from this emotional triad referring to motor aggression. Statistical analysis indicates that these comments were less present than expected (*residual adjustment* = −3.2). In this case, testimonies reported feeling joy despite receiving motor aggression actions from the hunters.

*“Joy because I was having a good time, even though I was being punished” C2*

This triad did not originate comments referring to motor interaction.

The emotional triad ON_GP_CP (yellow color) gave rise to 26 comments represented by negative emotional meanings in the role of Bear. Most comments (*n* = 19) expressed the discomfort originating from the motor aggressiveness (being hit with the scarf by the hunters). The statistical test shows more comments than expected (*residual fit* = 2.8).

*“I felt angry because of the blows I received" C3; "I felt fear because I did not want to be hit too hard” C4.*

This emotional triad only gave rise to 6 comments of emotional discomfort referring to the role (for playing an uncomfortable role in this game and being unable to change roles until the Guardian touched a Hunter). The statistical test tended to observe fewer comments than expected (*residual adjustment* = −3.1).

The emotional triad ON_GN_CP (orange colour) gave rise to the highest number of comments (*n* = 31), although the trends were not significant. Most comments were directed at motor aggression (*n* = 19; *residual fit* = 1.6) and role (*n* = 12; *residual fit* = −1.4).

*“Being hit, you are afraid of being hurt” C5.*

### Units of emotional meaning in the Guardian role

The 90 comments that originated from the Guardian role were related to the three dimensions of emotional meaning, i.e., role, motor interaction, and motor aggressiveness.

In the role dimension, the Guardian expressed a sense of well-being because of the attributes of this role and its dynamism in changing roles. On the other hand, some people expressed discomfort with the characteristics of this role (the Guardian has to keep an eye on the Hunters and the Bear).

In the motor interaction dimension, well-being was associated with the possibility of catching the Hunters or in the very act of catching him. It showed discomfort when the Guardian had difficulties in capturing a Hunter.

Concerning motor aggression, there was a predominance of comments of discomfort when faced with the responsibility of preventing the Bear from being attacked by the Hunters or for not being able to prevent it. The Guardian also expressed discomfort at not wanting to attack the hunters. Other people show well-being by preventing the Bear from being aggressed. It is observed that intervention in this role, and the game in general, favors well-being even if aggression is not avoided.

### Relationship of emotional meaning with the emotional triad in the Guardian role

In contrast to the Bear role, the 90 emotional comments in the Guardian role had a similar distribution across the three dimensions of emotional meaning: motor interaction (*n* = 37), motor aggressiveness (*n* = 27) and role (*n* = 26). Three emotional triads stand out, of which only one expresses emotional distress in the Guardian role (ON_GN_CP; orange color). The other two triads are associated with comments referring to the emotion of joy that participation in this role arouses (OP_GP_CP; blue color; ON_GP_CP; yellow color).

**Figure 9:**
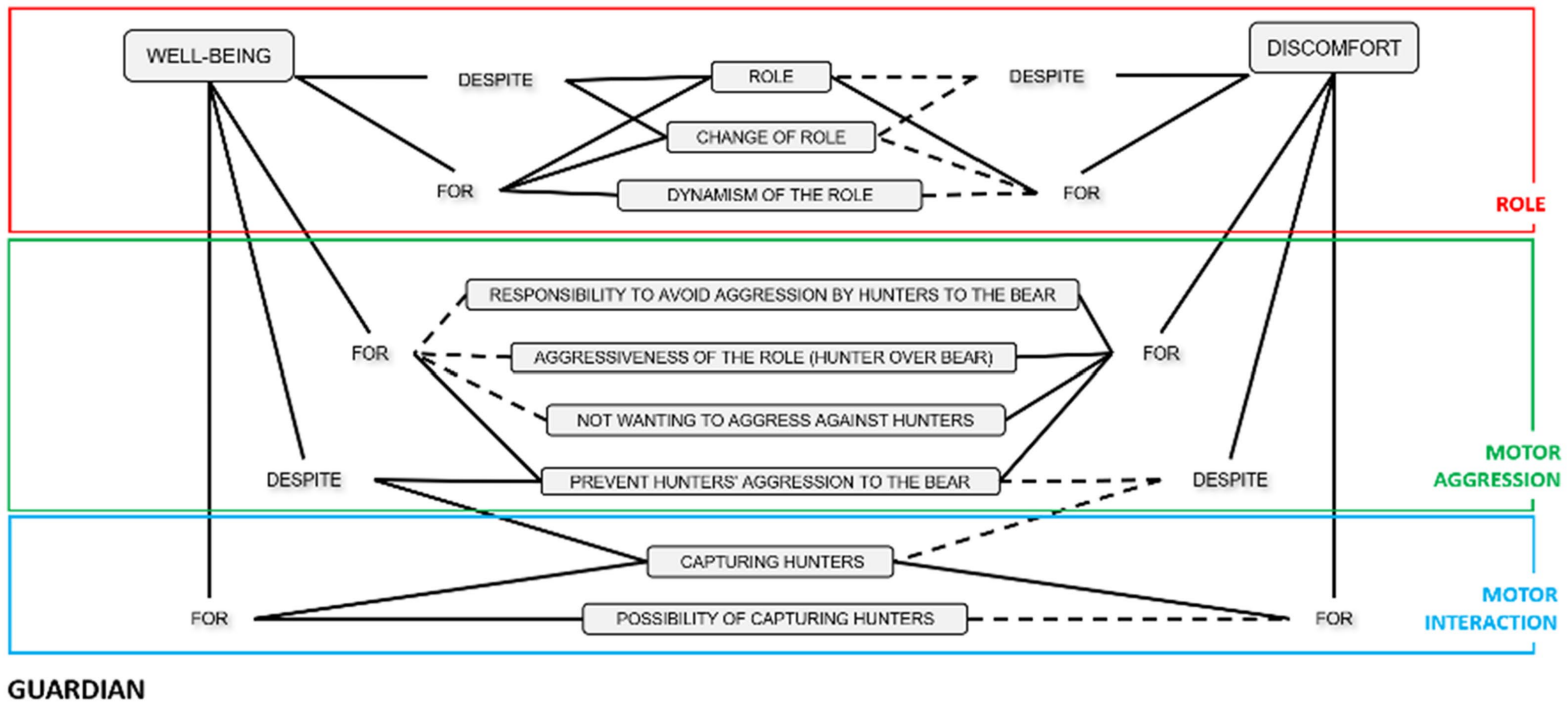
Orientation of emotional triads in the Guardian role.

The cross-table statistical test relating the three types of comments (role, motor interaction and motor aggressiveness) to the emotional triads found significant differences (*p* = 0.041; *Cramer’s V* = 0.41; see Figure 9).

The emotional triad ON_GN_CP (orange color) was associated with 31 comments referring to negative emotions in the role of Guardian. Significant differences were observed in words referring to motor interaction (*n* = 19; *residual adjustment* = 2.8). These comments described the discomfort of not being able to catch the hunters.

*“Sometimes I could not catch the hunters, and I felt bad “C5.*

Secondly, 10 comments referring to motor aggression were found. However, no significant differences were found (*residual fit* = 0.3). These comments referred to the discomfort of not wanting to attack the Hunters, not avoiding aggression toward the Bear and the responsibility of not avoiding aggression.

*“I felt anger at having to defend the person who has the role of the bear. In other words, if I didn't do my job properly, the person inside got hit a lot because of me”. C6*

Finally, there were fewer comments than expected, referring to the role (*n* = 2; *residual adjustment* = −3.4). These testimonies described emotional discomfort at being unable to change roles and being in a role they did not like.

The emotional triad ON_GP_CP (yellow) gave rise to 26 comments referring to the emotion of joy in the role of Guardian. Of these comments, significant differences were observed only in words referring to the role being more present than expected (*n* = 19; *residual adjustment* = 2.8). The testimonies highlighted the joy of the characteristics of this role, its dynamism and ability to change roles.

*“I felt joy at the thought of how close I was to be able to change my position to that of a hunter” C7.*

Thirdly, the emotional triad OP_GP_CP (dark blue) generated 21 comments on emotional well-being. Only the comments referring to motor aggression caused significant differences. A higher number of words were recorded than could be expected (*n* = 10; *residual adjustment* = 2.0). These comments highlighted joy at chasing or at the capture of the Hunters.

*“I felt joy because it was funny and amusing how the others were trying to run away so I would not hit them” C8*

### Units of emotional meaning in the Hunter role

The 90 comments collected in this role originated emotional meaning units referring to the three dimensions: role, motor interaction and motor aggressiveness.

In the role dimension, emotional well-being originated from this role’s characteristics and the strategy adopted.

In the motor interaction dimension, joy was present among the Hunters who avoided being captured by the Guardian and for opposing both the Guardian and the Bear as a team. Negative emotions originated from the possibility of being caught.

In the dimension of motor aggression, emotional well-being arose from attacking the Bear by observing how other Hunters beat him and his emotional reaction when he was assaulted. Emotional discomfort in this dimension arose due to the aggressiveness of the role of the Hunters on the Bear.

### Relationship of emotional meaning with the emotional triad in the Hunter role

The 90 emotional comments of the Hunter Role mainly referred to the emotional meaning dimension of the Role (*n* = 42), followed by the testimonies referring to motor aggressiveness (*n* = 36) and lastly to motor interaction (*n* = 12).

**Figure 10:**
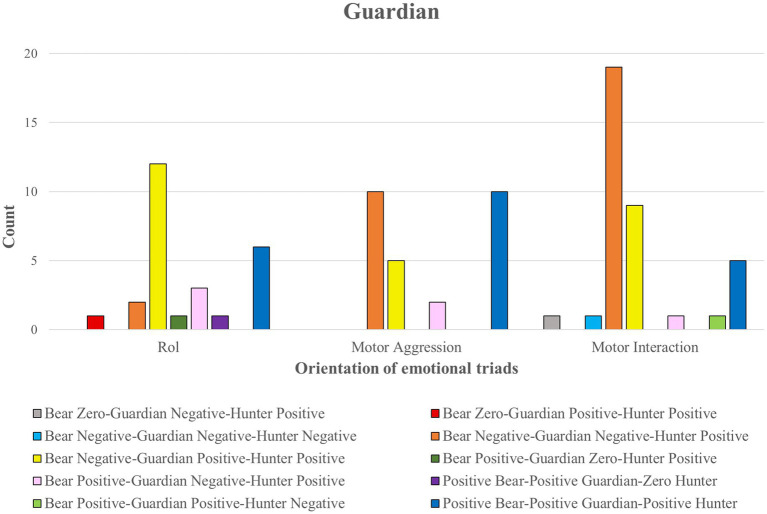
Orientation of emotional triads in the Hunter role.

As in the other roles, participants in the Hunter role experienced three emotional triads corresponding to joy. ON_GN_CP (orange color); ON_GP_CP (yellow color); and OP_GP_CP (blue color; see Figure 10).

The cross-table statistical test relating the three types of comments (role, motor interaction and motor aggressiveness) to the emotional triads showed significant differences (*p* = 0.046; *Cramer’s V* = 0.46) for the triad OP_GP_CP (dark blue color). This triad was associated with 21 positive comments, of which only six comments referring to the role were significant (*residual adjustment* = −1.9). The statistical test indicates that more comments were expected to have been recorded. These comments described joy about the characteristics of the Hunter role and satisfaction with the strategy adopted.

*“I was happy because it favored other teammates being able to attack the bear when I pushed the Guardian away”. C9*

Although no significant differences were found, 11 comments were recorded that expressed well-being when participating in motor aggression (by attacking the Bear, by observing how the Hunters attacked the Bear and their emotional reaction), and when the Guardian prevented aggression from other Hunters).

*“Because it was funny to see the reactions of the bear when we were going to hit him”.C10*

The emotional triad ON_GN_CP (orange) elicited the highest number of comments (n = 31). Although no significant differences were found, there was a tendency for emotional well-being to refer mainly to a role (*n* = 17) and motor aggression (*n* = 11).

*“The most intense emotion was joy because it was the most ‘fun’ role”. C11.*

The other emotional triad ON_GP_CP (yellow) behaved similarly to the previous one (*n* = 26). Although no significant differences were found, there was a tendency for emotional well-being to refer to role characteristics (*n* = 13) and motor aggression (*n* = 9).

*"It has given me much joy to be able to hit my partner in the middle" C12*

### The itinerary of emotional meanings in each of the three roles

Figure 5 shows the relationship between the emotional orientation of the three groups of comments (role, motor interaction and motor aggressiveness) for each role (Bear, Guardian, Hunter). The emotional meanings gave rise to emotional triads with a chained emotional orientation for each role (no value = OC; GC; CC; positive emotion: OP, GP, CP; negative emotion: ON, GN, CN). A greater thickness of the lines linking comments to emotions indicates greater frequency.

Each role gives rise to emotional singularities for the different dimensions of emotional meaning.

The role dimension elicited a thick line toward the three emotional options in each of the roles Bear (OC, ON, OP), Guardian (GC, GN, GP), and Hunter (CC, CN, CP).

The motor interaction dimension gave rise to a weak line of negative emotion in the Bear (ON). It elicited two thick lines of positive and negative emotion in the roles of Guardian (GP, GN) and Hunter (CP, CN).

The motor aggressiveness dimension projected three thick solid lines in the Bear role (OC, OP, ON), two thick lines of positive and negative emotion in the Guardian role (GP, GN) and one thick line of positive emotion in the Hunter role (CP).

### The emotional itinerary in the chained role changes

Figure 6 shows the emotional pathway expressed by the players during the game. The lines connect the type of emotion described in each role, and the emotional triad felt. The greater the thickness of the lines, the more frequently the connection occurred. The thickest links were ON_GN_CP (dark blue), ON_GP_CP (orange), and OP_GP_CP (green).

It can be seen that the most intense lines are associated with positive emotions that originated from a total of 17 branches (Bear = 3, Guardian = 3, Hunter = 3, and Triads = 7). This situation is followed by negative emotions with nine ramifications (Bear = 2, Guardian = 3, Hunter = 2, and Triads = 2). Finally, zero values originate five ramifications (Bear = 2, Guardian = 1, Hunter = 1 and Triads = 1).

### The association rules between emotional meanings and emotional triads

Using statistical procedures, we identified the 14 rules of association between the emotional meanings of the roles Bear, Guardian and Hunter, referring to the emotional meaning related to the role, motor interaction, motor aggressiveness and emotional triads (see Table 2).

**Table 2:** Pairwise association rules (premises and conclusion) for emotional meanings and emotional triads (according to support, confidence <0.04 and lift indicators).

No.	Premises	Conclusion	Support	Confidence	Lift
1	Bear motor aggression	Hunter role	0.211	0.422	0.905
2	Bear motor aggression	Bear negative-guardian negative-hunter positive	0.211	0.422	1.226
3	Bear motor aggression	Bear negative-guardian positive-hunter positive	0.211	0.422	1.462
4	Hunter role	Bear motor aggression	0.211	0.452	0.905
5	Bear motor aggression	Hunter motor aggression	0.233	0.467	1.167
6	Guardian motor interaction	Bear negative-guardian negative-hunter positive	0.211	0.514	1.491
7	Bear role	Hunter role	0.256	0.523	1.120
8	Bear motor aggression	Guardian motor interaction	0.267	0.533	1.297
9	Hunter role	Bear role	0.256	0.548	1.120
10	Hunter motor aggression	Bear motor aggression	0.233	0.583	1.167
11	Bear negative-guardian negative-hunter positive	Bear motor aggression	0.211	0.613	1.226
12	Bear negative-guardian negative-hunter positive	Guardian motor interaction	0.211	0.613	1.491
13	Guardian motor interaction	Bear motor aggression	0.267	0.649	1.297
14	Bear negative-guardian positive-hunter positive	Bear motor aggression	0.211	0.731	1.462

**Figure 11:**
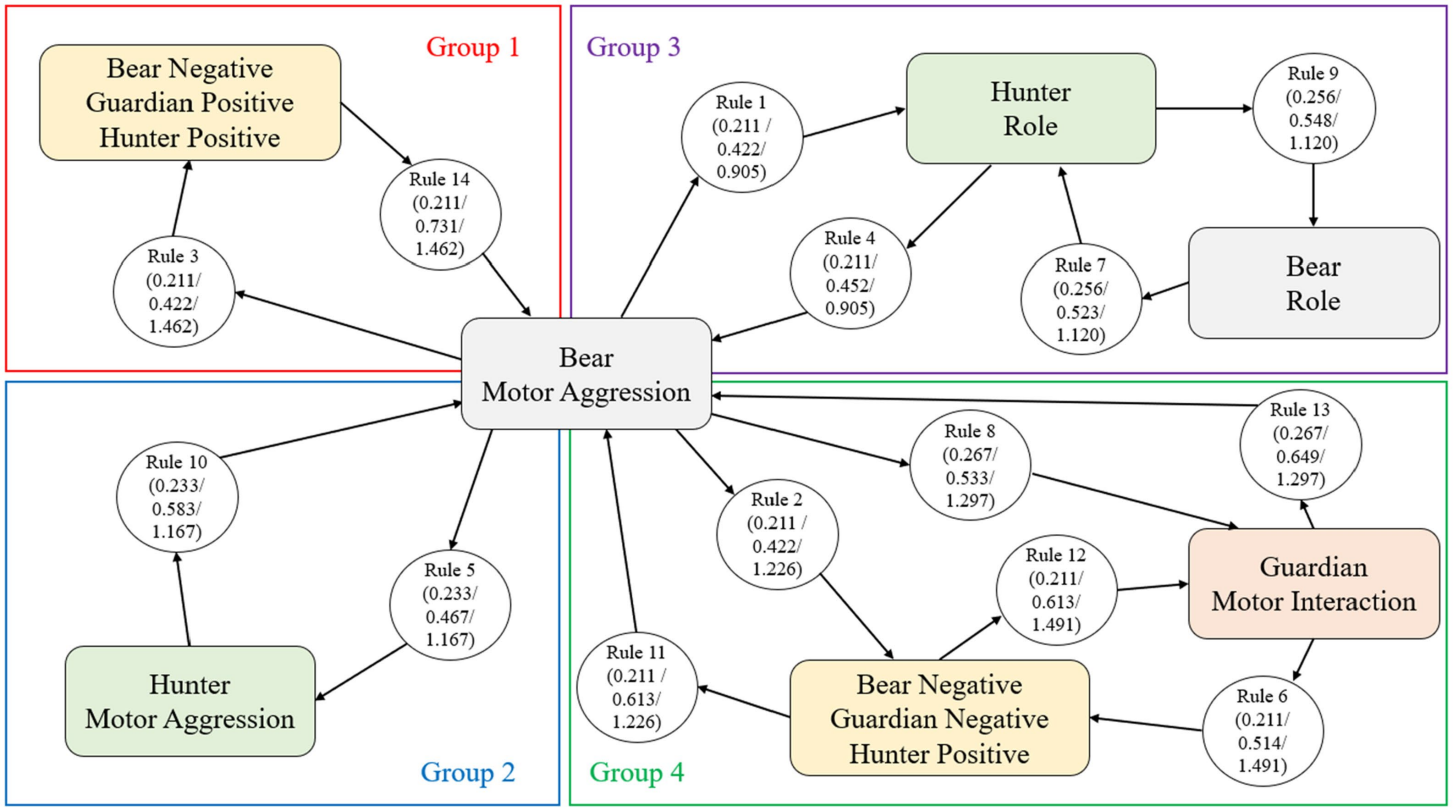
Emotional itineraries (groups of association rules) based on the emotional meaning of the Bear associated with Motor Aggression by support, confidence and lift measures.

Figure 11 highlights four significant groups of association rules with different meaning paths and emotional triads. In some of the highest values of support as a percentage of frequencies between the antecedent (premises) and the consequent (conclusion), confidence (< 0.04) corresponding to the effectiveness of the rule since the first requirement is met, that is, the appearance of an antecedent (premises) and lift (relative to the confidence of the rule concerning the consequent).

In the first emotional pathway (group 1 of association rules) of Figure 11, the emotional meanings of the Bear to aggression were linked to rule 3 (*confidence value* = 0.422; *high lift value* = 1.46), subsequently associated with the emotional triad Bear Negative-Guardian Positive-Hunter Positive. Finally, rule 14 (which obtained the highest confidence value of 0.731 and a high lift value of 1.46) was associated with motor aggression toward the Bear.

In the second emotional pathway (group 2 of association rules), the Bear’s emotional meanings of aggression were related to rule 5 (*confidence* = 0.47), which were then associated with the emotional meanings of the Hunters’ aggression. They were then associated with rule 10 (*confidence* = 0.58) and finally with the Bear’s emotional meanings of motor aggression.

In the third emotional itinerary (group 3 of association rules), the emotional meanings of the Bear to aggression were related to rule 1 (*confidence* = 0.422), then associated with the emotional meanings of the role-referred hunters. Two possible associations were then presented:

1. Through rule 9 (*confidence* = 0.548), and then with the emotional role-related meanings of the Bear. Then it was related to rule 7 (*confidence* = 0.523) to address the emotional role-related meanings of the hunters again.
2. Through rule 4 (*confidence* = 0.452), this itinerary returned to the Bear’s emotional meanings of motor aggression.

All the rules associations were connected in the fourth emotional pathway (group 4 of association rules). The emotional meanings of the Bear in the face of aggression were related to the Guardian concerning motor interaction through rule 8 (*confidence* = 0.533 and the highest *value of support* = 0.27) and rule 13 (which registered a high confidence value of 0.649 and the highest support value of 0.27).

Moreover, the Guardian concerning motor interaction was also related to the emotional triad Bear Negative-Guardian Negative-Hunter Positive by rule 6 (*confidence* = 0.514; *lift* = 1.491and rule 12 (with a high confidence value of 0.613; with a high lift value of 1.49).

The Bear Negative-Guardian Negative-Hunter Positive emotional triads were connected by the Bear’s emotional meanings of aggression by rule 11 (*confidence* = 0.613; *lift* = 1.226) and rule 2 (*confidence* = 0.211; *lift* = 1.226).

## Discussion

The present research aimed to study the emotional intensity in the roles of Bear, Guardian and Hunter, as well as the units of emotional meaning and their correspondence with the emotional triad in the three roles.

The data obtained confirm that the ritual order that establishes the motor interactions of this game is associated with the constant chained change of roles, which also involves the relationships between the players and their emotions.

The first finding shows that in all three roles, the players experience, above all, positive emotions. However, statistical evidence reveals that each role carries unequal emotional experiences. Positive emotions achieve the highest intensity in the Hunter role and less in the Bear role.

Negative emotions are highest in the Bear role and lowest in the Hunter role. The Guardian occupies an intermediate position, both in positive and negative emotions.

To contextualize and interpret this first finding, it is it is necessary to go beyond the quantitative data. Thus, to unveil the meaning of emotional intensity, it is necessary to know why players feel different intensities of emotions in this ritual of singular motor interactions. Hence, in the content analysis of the players’ testimonies in the three roles, emotional meanings associated with the role, motor interaction, and motor aggressiveness have been identified. In addition, using other statistical tests leads us to affirm that each role contains unique socio-affective traits intertwined with the rest of the roles. The Bear, the Guardian and the Hunter originate a chained role change, in which the three roles provide feedback, need each other and complement each other in this socio-affective warp of interdependent relationships. Hence the interest in considering the emotional triads as an indissoluble unit of the effects of the game.

The analysis of the testimonies proves that the relationship with time is one of the critical pillars on which the internal logic of this game rests (Parlebas, 2001). The game leads the participants to change roles and relationships constantly. It is an interactive ritual in which who was a friend becomes an enemy or vice versa.

Unlike sports, the rules of this game do not establish how it ends. The game gives rise to a cyclical, non-causal, purposeless time (Rosenblueth et al., 1943), an open time (Suits, 1978). There is no final scoreboard, and there are no winners or losers, so when the game ends, the participants will not be ordered hierarchically, as in sports (Etxebeste et al., 2014).

The emotional meanings for each role show that well-being or discomfort may be associated with remaining more or less time than desired in each of these roles, i.e., that the temporal flow of the game, with greater or lesser speed, originates the dynamics of role changes.

### The role of bear. Well-being subordinated to socio-affective power relations

Statistical evidence reveals that the Bear plays the central role in the game. Emotional meanings refer to motor aggression working like a magnet that attracts four itineraries of rules of meaning association and emotional triads.

This role cannot generate motor interactions, as is evident from the virtual absence of comments on this dimension. The Bear is sitting on the floor with a passive attitude. Its emotional states depend on the following: (a) the speed with which the change of role occurs, (b) the intensity of the motor aggressions received by the Hunters, and (c) the protective efficacy of the Guardian.

Playing the role of the Bear involves receiving motor aggression and experiencing triads of negative emotions in this role while simultaneously eliciting positive emotions in the Guardian and the Hunter. When the emotional significance is directed exclusively at the Bear role, the rest of the players, in general, can experience positive emotions, contextualizing this fact in the course of the game.

Going through this role requires a good dose of resilience. An opportunity to learn to adapt intelligently to the adverse situations and threats this role receives, putting the necessary self-esteem, courage, hope and patience to the test.

*“Because I knew I would only be there for a short time, I would close my eyes and listen to the laughter of my colleagues in the hope of changing roles” C13.*

The Bear also needs to adapt to accept with resignation the beatings he/she receives from the Hunters, as the rules do not allow him to intervene.

*“I felt discomfort as they are hitting you, and you cannot fight back” C14.*

Finally, the Bear needs to cultivate trust and hope with the Guardian. This role generates the expectation that the Guardian will protect her/him, according to the internal logic of this game, with an attitude of respect, understanding and generosity.

*In the role of the Bear, I was hoping that the Guardian would not take too many risks to catch the Hunters and, therefore, not receive too many hits.C15*

### The Guardian role. Well-being in the face of the responsibility to orientate motor interactions toward power and/or status

In the role of Guardian, 12 units of meaning have been identified, of which the majority (67%) refer to relationships with others (8), referred to motor aggressiveness (5) and motor interaction (3).

The testimonies indicate that the Guardian is the most dynamic role with the highest level of responsibility for the players, as he is the only player who, by being effective in his actions, will provoke a change between the three roles.

*“You feel a bit of adrenaline and excitement of wanting to get everywhere, and no one touches the Bear and continually making decisions”. C16*

When adopting an empathic strategy with the Bear, the Guardian engages in “status” interactions (Kemper, 1981) to protect the Bear. She/he weighs up the risk to the Bear in his/her relationship with the space. The Guardian chooses to move just far enough away from the Hunters closest to the Bear to come back when they are attacking her/him or very close to her/his position. Moreover, when he/she can hit a Hunter, he/she does not do so with intensity. The game’s internal logic activates socio-motor empathy processes (Parlebas, 2001), which involves abandoning one’s point of view and putting oneself in the place of the other participants. Empathic motor conduct includes a cognitive load (appreciation of distances, estimation of chances of success), and an affective engagement (perception and management of emotions in a risky situation) (Parlebas, 2001).

*“I was happy because I caught it quickly, and my Bear hardly got hit” C17.*

The burden of responsibility, coupled with a lack of effectiveness (self-relatedness), intense beatings of the Bear (Hunter-Bear relationship) and intense motor aggression on a Hunter (Guardian-Hunter relationship), can cause emotional distress.

*“Anger because the Hunters were attacking the Bear, and that did not seem right to me. I wanted to avoid that situation because I was giving an advantage to Hunters to hit the Bear” C18.*

On other occasions, the Guardian engage in social rituals of power (Kemper, 1981), prioritizing their interests (wanting to change roles) and the will to dominate (capture) the Hunters. This orientation can lead to prioritizing their interests, even if it means exposing the Bear’s protection and decreasing levels of solidarity or sociomotor empathy (Parlebas, 2001).

*“It was fun; you were just worried about catching people”. C19*

*“I had to hit my partners so I could change roles; that made me happy” C20*

### The role of hunter. The pleasure of starring in power motor interactions

The role of the Hunter is who gives rise to the greatest number of emotional triads of well-being, even if they involve negative emotions for the Bear and the Guardian. Players like this role in general and the variety of strategies it elicits and can be adopted.

This role is responsible for the game’s main social motor power interactions (Kemper, 1981). Each Hunter decides the level of motor aggression that will or will not punish the Bear.

This role tests Hunter’s civilizing process of self-control of emotions (Elias, 1987), whose success (well-being) is associated with the Bear’s failure and pain (discomfort). After deciphering the exchange of emotional meanings with the others, the Hunter can decide whether to try to dominate the Bear and the Guardian or to seek an exchange of motor interactions that satisfies his/her needs and those of the others.

*“The most intense emotion was fear, as I was afraid of hitting the Bear too hard and hurting him/her”.C21*

In this role, people have to channel violent and aggressive behaviors and can transform them into cordial and respectful relationships. Aggressive motor conduct (Collard, 2004; Collard and Oboeuf, 2007; Dugas, 2008) can be transformed into care, protection and respect for the person in the Bear role (Loyer et al., 2015). Players can promote moral order through motor behaviors that are honorable, dignified and respectful of this ludomotor encounter (Goffman, 2004; Parlebas, 2016).

When power interactions predominate, some Hunters feel emotional well-being when attacking the Bear. When power interactions predominate, some Hunters feel emotional comfort in attacking the Bear. It has been observed that some players hold the scarf by both ends with both hands, twisting it in circles to tighten the handkerchief and hit the Bear hard.

Some people express well-being by acting as a team, adopting a collective strategy. This situation involves changing the motor communication network presented by the game’s rules. It moves from the confrontation Team against the rest (Bear-Guardian vs. Hunters) to a confrontation between two teams (Team Bear-Guardian vs. Team Hunters; Parlebas, 2001). The superiority and dominance of the Hunters over their opponents, i.e., the interaction of power is accentuated.

*“I was happy to be a hunter because I had the same goal as my colleagues, and we could collaborate or help each other. Having a ‘team’ made me feel happy”.C22*

Other Hunters may orient their motor behaviors toward status motor interactions. In these circumstances, the emotional meaning is a testament to their sociomotor empathy.

*“I felt happy because I could hit my colleagues in a friendly way without hurting them”. C23*

Often, the Hunter expresses discomfort at the aggressiveness of some Hunters toward the Bear.

The hunters showed little empathy for the person being whipped (the Bear). Some people did not control the force or the place where they hit (face, for example) and were going to harm.

Equally, there is joy in observing that the Guardian avoids motor aggression toward the Bear. Moreover, in lighthearted situations, the Bear’s emotional reaction (e.g., laughing or shouting jocularly) can also be a source of satisfaction.

*“Because I was amused to see the Bear's reactions when we were going to hit him/her”.C25*

## Conclusion

The Game of Bear-Guardian and Hunters is an authentic laboratory of interpersonal relations and emotions (Parlebas, 2001). It is a traditional sporting game associated with a unique motor communication network.

This game contains a ritual that organizes and orders the relationships between people and how emotions are expressed and managed. Players share a constant flow of emotional energy. When the plot is cordial and pleasurable, people’s emotions enter into states of reciprocal consonance (Collins, 2009). In this way, the practice of this traditional game triggers the literacy of “feeling rules” (Hochschild, 1979).

It is a game that contains several games or sets of socio-affective experiences for each role. At the end of the game, each person takes away a set of subjective emotional meanings that give rise to unequal emotional triads, depending on their intervention in this web of interlinked roles. Each person prioritizes social motor interactions of status or power during the game. Resilience, confidence, and resignation, combined with protection, social-motor empathy and mastery, are processes that people need to adapt to and internalize to play.

The methodology revealed part of this game’s secret code (intimate and subjective). The affectivity invisible to external observation can be unveiled through the testimonies of the participants. The comments confirm that this game, if used well, can be an extraordinary tool to foster quality physical education (UNESCO, 2016) and to promote sustainable development (United Nations (UN), 2015). Players must adapt to a holistic understanding of their own and others’ well-being to play better.

The time that runs in a role depends fundamentally on the others and the motor interaction they decide on; it will trigger the continuation of the actors’ itinerary, which is determined by the game’s internal logic. Based on a spatio-temporal relational ritual, the role changes in the game of the Bear-Guardian and Hunters thus appear as true revelators of the socio-emotional experience.

People participating as players through their motor conduct systemically activate their whole personality. When playing, each person attaches unique meanings to the decisions, relationships, emotions and organic aspects of playing each role.

After each change of role, a new adventure begins, a new project and a new web of meanings of motor interactions of status or power (Kemper, 1981). The social ritual starts all over again, and this is how the players reinstate a particular way of relating to each other as people did several centuries ago. We are dealing with a distinct culture expressed through motor conduct. Bodies are cultural signs that teach how to live in a society (Parlebas, 2001), in this case, with the possibility of improving the way of living together.

**Figure 12:**
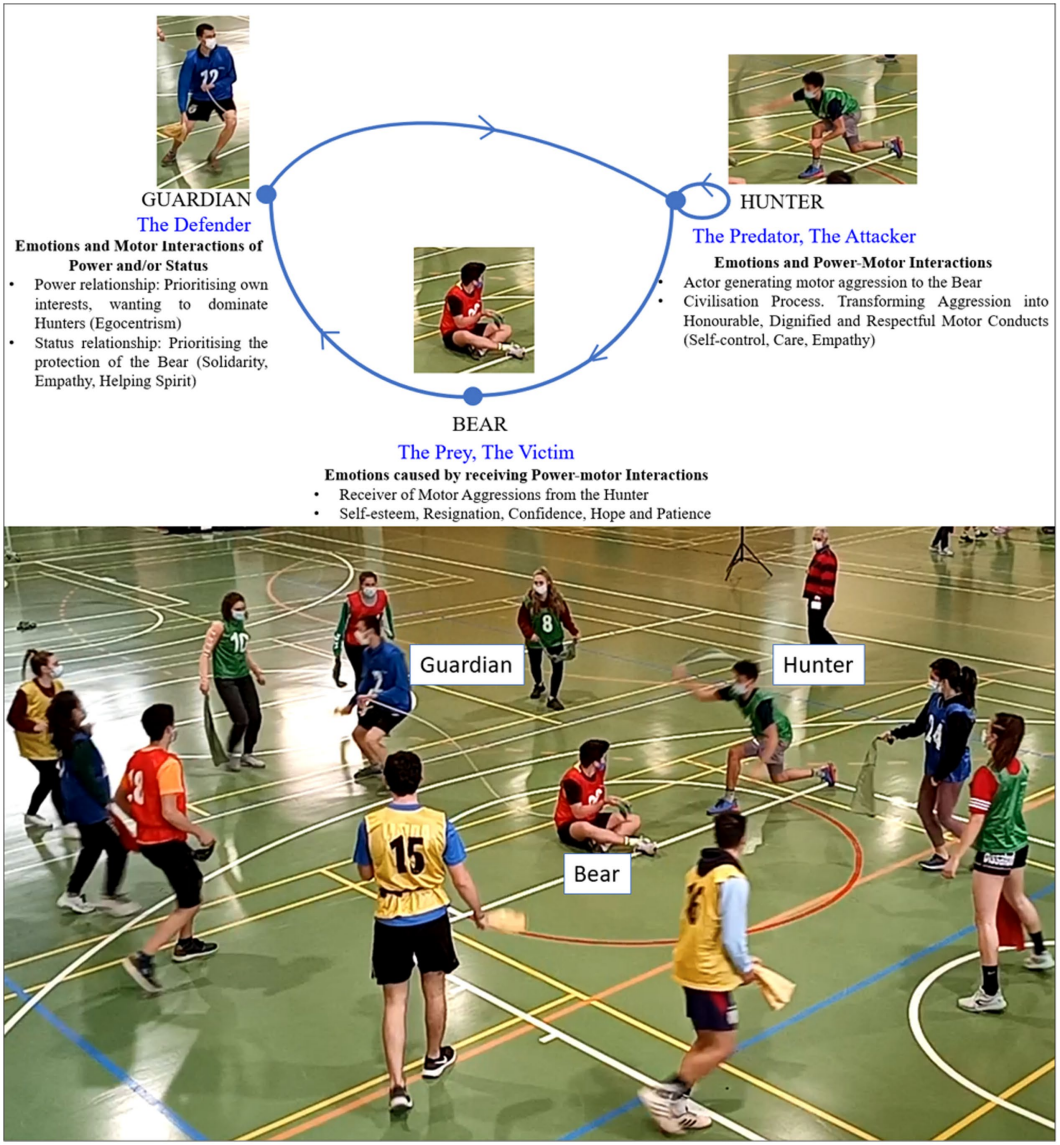
The chained role changes network. Emotions and status/power motor interactions. The line represents the change of role. The dot corresponds to the role. The loop over the Hunter role shows that when a role change occurs, some players remain in the same role, as only one hunter changes to the Bear role.s

The Bear, Guardian and Hunters is a proper theater of life in which the actors play and act as a whole, each person integrating meaning into each of the three roles in which he or she is involved (see Figure 12).

With its historical roots going back many centuries, this game offers a perspective that punctuates the civilizing processes (Elias, 1987) followed by humanity. Over the years, the motor aggression of the Hunter over the Bear role has been tempered, civilized, and softened in a process that is not yet finished. Right now, this game in the hands of intelligent, prepared and sustainable teachers could help this democratic process to continue advancing this process of civilization (Elias and Dunning, 1994).

The limitation of this research is that it is based exclusively on the subjective testimonies of the participants. It would be advisable to complement these findings with observational methodology and use other instruments (e.g., accelerometers or GPS) to analyze other dimensions to confirm, contextualize and complement the results obtained.

## Data availability statement

The raw data supporting the conclusions of this article will be made available by the authors, without undue reservation.

## Ethics statement

The studies involving human participants were reviewed and approved by Ethics Committee for Clinical Research of the Catalan Sports Council (07/2019/CEIGEGC). The patients/participants provided their written informed consent to participate in this study. Written informed consent was obtained from the individual(s) for the publication of any identifiable images or data included in this article.

## Author contributions

PL-B, VA-M, CM-L, and MP: substantial contribution to study conception and design, discussion of data analysis strategies, and writing of the manuscript. PL-B and CM-L: preparation of the document for approval by the ethics committee, and preparation and participation in the empirical work. PL-B, VA-M, and CM-L: content analysis. MP: statistical analysis and preparation of the databases (all variables). All authors contributed to the article and approved the submitted version.

## Funding

This work was supported by the National Institute of Physical Education of Catalonia (INEFC; code: DOGC N. 8568–22.12.2021 Resolution 21_03_2022) and cofounded by the European Commission Erasmus+ Opportunity Project. Code: 622100-EPP-1-2020-1-SE-SPO-SCP).

## Conflict of interest

The authors declare that the research was conducted in the absence of any commercial or financial relationships that could be construed as a potential conflict of interest.

## Publisher’s note

All claims expressed in this article are solely those of the authors and do not necessarily represent those of their affiliated organizations, or those of the publisher, the editors and the reviewers. Any product that may be evaluated in this article, or claim that may be made by its manufacturer, is not guaranteed or endorsed by the publisher.
